# Exploring Powered Wheelchair Users and Their Caregivers’ Perspectives on Potential Intelligent Power Wheelchair Use: A Qualitative Study

**DOI:** 10.3390/ijerph110202244

**Published:** 2014-02-21

**Authors:** Dahlia Kairy, Paula W. Rushton, Philippe Archambault, Evelina Pituch, Caryne Torkia, Anas El Fathi, Paula Stone, François Routhier, Robert Forget, Louise Demers, Joelle Pineau, Richard Gourdeau

**Affiliations:** 1 School of Rehabilitation, Université de Montréal, C.P. 6128 Succursale Centre-Ville, Montréal, QC H3C 3J7, Canada; E-Mails: paula.rushton@umontreal.ca (P.W.R.); evelina.pituch@umontreal.ca (E.P.); louise.demers@umontreal.ca (L.D.); 2 Center for Interdisciplinary Research in Rehabilitation (CRIR)—IRGLM Site, 6300 Avenue Darlington, Montréal, QC H3S 2J4, Canada; E-Mail: robert.forget@umontreal.ca; 3 Centre de Recherche de l’Institut Universitaire de Gériatrie de Montréal (CRIUGM), 4545 Chemin Queen Mary, Montréal, QC H3W 1W4, Canada; E-Mail: pstone@videotron.ca; 4 School of Physical and Occupational Therapy, McGill University, 3654 Promenade Sir-William-Osler, Montréal, QC H3G 1Y5, Canada; E-Mail: caryne.torkia@mail.mcgill.ca; 5 Center for Interdisciplinary Research in Rehabilitation (CRIR)—JRH Site, 3205 Place Alton-Goldbloom, Laval, QC H7V 1R2, Canada; E-Mail: philippe.archambault@mcgill.ca; 6 Polytechnique Montréal, C.P. 6079 Succursale Centre-Ville, Montréal, QC H3C 3A7, Canada; E-Mails: anas.el-fathi@polymtl.ca (A.E.F.); richard.gourdeau@polymtl.ca (R.G.); 7 Department of Rehabilitation, Faculty of Medicine, Université Laval, Pavillon Ferdinand-Vandry, 1050 Avenue de la Médecine, Québec, QC G1V 0A6, Canada; E-Mail: francois.routhier@rea.ulaval.ca; 8 Center for Interdisciplinary Research in Rehabilitation and Social Integration (CIRRIS), Institut de Réadaptation en Déficience Physique de Québec (IRDPQ), 525 Boulevard Hamel, Québec, QC G1M 2S8, Canada; 9 School of Computer Science, McGill University, 3480 University Street, Montréal, QC H3A 0E9, Canada; E-Mail: jpineau@cs.mcgill.ca

**Keywords:** intelligent power wheelchair, mobility, obstacle-avoidance, path following, navigation, user-centered design, disability, safety

## Abstract

Power wheelchairs (PWCs) can have a positive impact on user well-being, self-esteem, pain, activity and participation. Newly developed intelligent power wheelchairs (IPWs), allowing autonomous or collaboratively-controlled navigation, could enhance mobility of individuals not able to use, or having difficulty using, standard PWCs. The objective of this study was to explore the perspectives of PWC users (PWUs) and their caregivers regarding if and how IPWs could impact on current challenges faced by PWUs, as well as inform current development of IPWs. A qualitative exploratory study using individual interviews was conducted with PWUs (n = 12) and caregivers (n = 4). A semi-structured interview guide and video were used to facilitate informed discussion regarding IPWs. Thematic analysis revealed three main themes: (1) “challenging situations that may be overcome by an IPW” described how the IPW features of obstacle avoidance, path following, and target following could alleviate PWUs’ identified mobility difficulties; (2) “cautious optimism concerning IPW use revealed participants” addresses concerns regarding using an IPW as well as technological suggestions; (3) “defining the potential IPW user” revealed characteristics of PWUs that would benefit from IPW use. Findings indicate how IPW use may help overcome PWC difficulties and confirm the importance of user input in the ongoing development of IPWs.

## 1. Introduction

Recent estimates from the World Health Organization indicate that some 65 million people worldwide need a wheelchair [1]. Statistics Canada reported in 2000–2001 that 264,000 people used wheelchairs as a primary means of mobility [2] and the United States Census conducted in 2010 reported that there were 3.6 million wheelchair users over the age of 15 [3]. In 2002 in the United States, there were 2.7 million non-institutionalized users of wheeled mobility devices, approximately 30% of which used powered wheelchairs (PWCs) or scooters [4]. Similar data has been reported for Europe [5,6]. Furthermore, power mobility device use will likely continue to increase given the growing prevalence of disability worldwide due to changing demographics such as an ageing population and an increase in chronic health conditions [7]. It is therefore essential to ensure that mobility devices, in particular powered mobility devices, best meet wheelchair users’ needs in order to facilitate participation and enhance quality of life. 

The benefits of power mobility, including improved self-esteem [8], decreased pain [9], and increased activity levels and social participation [10,11,12,13] are well documented. PWC use also has its challenges. For example, power wheelchair users (PWUs) report being afraid to navigate in crowded spaces with their device [14]. In addition, clinicians who prescribe wheelchairs report that some clients cannot use PWCs safely because of visual, motor and cognitive deficits [15,16,17]. Smart or intelligent power wheelchairs (IPW) that provide navigation assistance have hence been proposed for PWUs who either cannot use, or have difficulty using, existing power mobility devices. These types of IPWs are not yet commercially available for use outside the lab and are still in the development phase. However, a review of studies looking at IPWs that provide navigation assistance to the PWU reports that these new mobility devices could benefit people with severe motor, sensory or cognitive limitations, allowing them to carry out their everyday activities [17]. Including users in the design process of new health technologies is increasingly recognized as essential in order to understand and consider users’ needs [18]. Including the user in the design process provides information regarding the needs, experience and ideas of future users, and has been found to lead to greater functionality, usability and quality of the devices that are developed [19]. User input at the prototype stage is essential so that design changes can be made prior to the manufacturing stage [20]. 

The objective of this study was to explore the perspectives of PWUs and their caregivers regarding IPW to better understand if and how IPWs could impact on current challenges faced by PWUs, as well as inform current development of IPWs.

## 2. Methods

### 2.1. Study Design

A qualitative exploratory study was used, with semi-structured individual interviews conducted with PWUs’ and their caregivers’, in order to obtain their perspectives regarding IPW use. 

### 2.2. Participants

Using a convenience sample, 12 PWUs and four caregivers were recruited for this study from the wheelchair and seating departments of two rehabilitation centers in Montreal. PWUs were included if they: (1) had been using a PWC in the community for at least one year, (2) were 18 years of age or older, (3) were able to express themselves in French or English, and (4) had any musculoskeletal or neurological diagnosis resulting in a long-term severe mobility limitation. PWUs were excluded if: (1) they had a communication difficulty and/or a hearing or vision deficit significantly limiting their ability to participate in the interviews, and (2) if they presented emotional or psychiatric problems or cognitive disabilities that could limit their participation in the study, as discussed with the participant’s referring therapist. 

Caregivers were recruited if: (1) they were informal caregivers or long term companions of a PWU and (2) they provided assistance to or accompanied a PWU in activities that involved the PWC, such that they could provide meaningful insight into current PWC use and possible IPW use. Caregivers did not have to be caregivers to the PWU participants in this study. 

### 2.3. Data Collection and Analysis

Individual interviews were conducted with PWUs and caregivers. Prior to the interviews, socio-demographic and PWC use information was collected using a sociodemographic form to document self-reported personal data (e.g., age, sex, diagnosis) and wheelchair data (e.g., duration of power wheelchair use, method of power wheelchair control, *etc.*) for PWUs. A different form was used to collect sociodemographic data from the caregiver (e.g age, sex, relationship to the PWU, extent of help provided with the PWC, *etc.*). A semi-structured interview guide with open-ended questions and probes was developed by the research team. This guide was modified as the interviews progressed in order to capture data about emerging themes. Participants were asked about past and current PWC use, including positive aspects (e.g., benefits, activities performed) and challenging aspects (e.g., barriers encountered, safety concerns, accidents). PWUs were also asked to describe any unmet mobility needs. All participants were then shown a four-minute video illustrating the functionalities of an IPW to facilitate informed discussion about the IPW and its relevance to them. This video illustrated the main features of a prototype IPW that our research team is developing (see section 2.5), used within the environment of a major shopping center in downtown Montreal (Quebec, Canada). After the video, participants were asked questions about their perception of the IPW (e.g., use, safety, confidence and relevance), as well as more specific questions about the relevance of the IPW features (e.g., path following, obstacle avoidance, target following). Caregivers were asked questions regarding PWC and IPWs with respect to both their role as a caregiver and what they perceived to be the impact on the PWU. The interviews were conducted in the PWU’s home by an occupational therapist with extensive knowledge of and experience with power mobility and the wheelchair community. Prior to meeting the participants (PWU and caregivers), she was aware of the PWU’s primary diagnosis and how long they had been using a PWC. When possible, separate interviews were conducted for the PWU and their caregiver. Interviews, conducted in English or French (depending on the participants’ preference), were audio recorded and transcribed verbatim in the language of origin. 

Data collection and preliminary analysis were conducted concurrently. Each interview was initially analyzed for general impressions by four team members (Dahlia Kairy, Paula W. Rushton, Evelina Pituch and Paula Stone) and initial codes were generated collaboratively [21]. Once each interview was analyzed individually, a more in-depth analysis of the codes across interviews was conducted and overarching themes were identified by the first two authors (Dahlia Kairy and Paula W. Rushton) using NVivo 8 software (QSR International, Doncaster, Victoria, Australia). Any differences in opinions regarding codes were resolved through discussion among team members involved in the data analysis process.

The study was approved by the Research Ethics Committee of the institutions managing the Centre for Interdisciplinary Research in Rehabilitation of Greater Montreal (CRIR) and informed consent was obtained from all participants prior to the interview.

### 2.4. Description of IPW Prototype Used for Video

**Figure 1:**
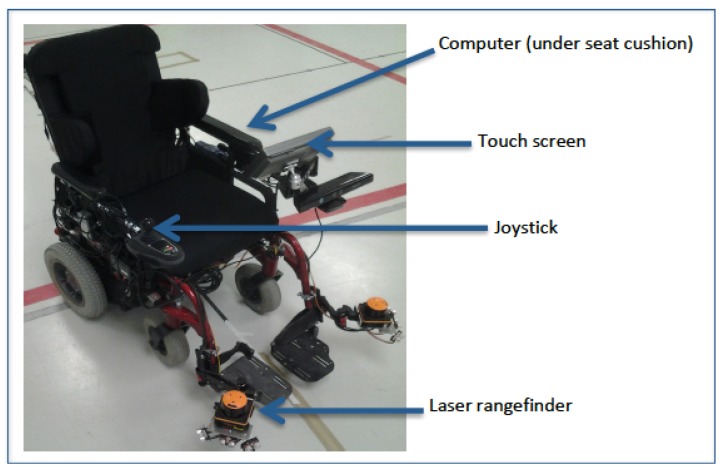
Intelligent power wheelchair prototype.

**Figure 2:**
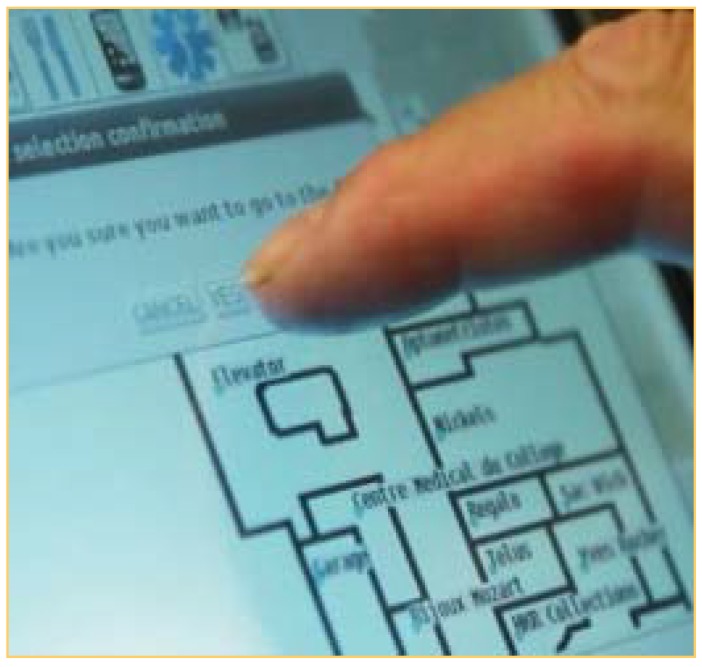
Tactile interface illustrating intelligent power wheelchair path finding function.

Since 2006, our multidisciplinary team has been developing a prototype IPW with semi-autonomous navigation functions, using robotic and artificial intelligence technologies (see Figure 1). The robot and computer interface, built onto a commercially-available PWC, can be controlled by speech recognition, a joystick or a tactile display (see Figure 2). Using laser and sonar sensors mounted on the chair, the IPW has several unique functions: (1) it can follow a planned path (path following); (2) it avoids static and dynamic obstacles (obstacle avoidance); (3) it negotiates through doorways and in between obstacles (path following/obstacle avoidance combination); (4) it can follow a given object such as a wall or a person or a group of people (target following). The IPW user has the choice to control the IPW as a regular PWC or to allow the “intelligent” functions to guide the chair [22]. The video used during the interviews illustrated the use of the IPW by a PWU in a mall setting, highlighting the intelligent functions of the chair.

## 3. Results

### 3.1. Participants and Interviews

Participant demographics are presented in Table 1. With regard to PWUs, there were twice as many men as women with ages ranging from 22–88 years. The primary diagnoses were neurological in nature and participants had used PWCs for at least 3 years. Three of the four caregivers lived with the PWU. Half of the PWUs did not need any assistance to navigate with their PWC, while five out of 12 needed help with transfers. All caregivers assisted the PWU in their activities, although the amount of help provided by the caregiver with respect to the PWC ranged from rarely to several times a week. 

**Table 1:** Participant characteristics.

Participant Characteristics	PWUs (n = 12)	Caregivers (n = 4)
Age (years), mean ± SD	55 ± 21	67 ± 10
Range	22–88	62–79
**Sex (n)**		
Female	4	0
Male	8	4
**Mother Tongue (n)**		
French	9	3
English	1	1
Other	2	0
**Primary Diagnosis (n)**		
*Musculoskeletal (25%)*		
Rheumatoid arthritis	1	
Osteoarthritis	1	
Fibromyalgia	1	
*Neurological (75%)*		
Parkinson’s disease	1	
Multiple sclerosis	4	
Spinal cord injury	1	
Muscular dystrophy	2	
Spinal muscular atrophy	1	
Duration of PWC use (years), mean ± SD	14 ± 12	
Range (years)	3–39	
**Location of PWC use (n)**		
At home	9	
At work/volunteer	5	
At school	3	
In the community	12	
In a shopping center	11	
Recreation/Sports	10	
**Method of current PWC control (n)**		
Joystick	7	
Head Control	2	
Other specialized control system	3	
**Level of assistance required with the PWC (n)**		
None	6	
Supervision	1	
Physical assistance	0	
Assistance with transfers	5	
**Relationship to PWU (n)**		
Spouse		2
Friend		2
Caregiver living in same residence as PWU (n)		3
**Frequency of help provided by the caregiver to the PWU, for activities related to PWC only (n)**		
Rarely		1
Once a day		1
Several times a week		1
Unknown		1

Interviews lasted on average 40 min (range 24–66 min) with the PWUs and 43 min (range 31–66 min) with the caregivers. One interview was conducted with a PWU who had severe dysarthria as a result of cerebral palsy. However, since the caregiver assisted the PWU in expressing his thoughts he was included in the study and interviewed with the caregiver.

### 3.2. Findings

When considering current PWC use and potential IPW use, 3 main themes emerged from the participants’ perspectives: (1) challenging situations that may be overcome by an IPW; (2) cautious optimism concerning the IPW; (3) defining the IPW user. These are presented in the following sections with verbatim quotes to illustrate the themes where appropriate. When needed, selected quotes have been translated from French to English by bilingual members of the research team for the purpose of presenting the results.

#### 3.2.1. Challenging Situations that may be Overcome by an IPW

PWUs described the “autonomy”, “independence”, and “freedom” that their PWC afforded them. The meaning of their PWC was expressed by PWUs in a variety of ways, including “The chair is my legs!” (59 years old, female, primary diagnosis of rheumatoid arthritis, 11 years as a PWU) and “I go everywhere with it!” (81 years old, male, primary diagnosis of fibromyalgia, 6 years as a PWU). However, PWUs and caregivers also recounted challenging situations that occurred with the PWC, as presented in Table 2. These are most commonly related to the built environment (e.g., inaccessible buildings, narrow entrances, small elevators, narrow store aisles, spaces made narrow by displays and temporary set ups), outdoor environment (e.g., poor visibility of the PWC user on the street, sidewalks in poor conditions, snow and rain, long distances), and crowded places (e.g., stadiums, festivals, malls). Some of the challenges are related to the physical impairments of the PWC user. For example, a common difficulty expressed by participants was backing up in the PWC such as when exiting a narrow elevator, in particular for participants with limited head mobility or control. 

For many of the challenging situations, PWUs and caregivers described strategies that had been developed to overcome the challenges, namely avoiding difficult situations, finding alternative activities or locations to perform the activities, or relying on the caregiver or other people in the area for assistance. Some strategies compromised the safety of the PWU, such as using reserved bus or bicycle lanes instead of sidewalks. Participants described a range of accidents and incidents that occurred as a result of their identified challenging situations, ranging from scraping their knuckles or banging doorframes to getting hit by cars or buses when not seen at crosswalks or in bus lanes.

After viewing the IPW video, participants discussed how the various IPW features would alleviate many of the challenging situations they had identified when using their current PWC (Table 2). Situations they described suggest context in which IPW use could be beneficial. Obstacle avoidance was identified as a feature that would minimize a number of difficulties frequently encountered in the physical environment, as highlighted by this participant’s statement, “…the fact that it can really calculate and see the distance and able to get through (doorways), it helps a lot for somebody who has difficulty in controlling the chair in narrow places.” (33 years old, male, primary diagnosis of muscular dystrophy, 11 years as a PWU). This feature was also perceived as useful to exit crowded or narrow elevators while backing up. Similarly, PWUs and caregivers described obstacle avoidance as a feature that would facilitate negotiating crowded spaces. For example, “avoiding pedestrians would be super in the (*hockey arena*)” (66 years old, male, primary diagnosis of cerebral palsy, 29 years as a PWU). Similarly, a PWU stated “Excellent. Excellent all these things (the IPW features), I find it interesting…because if a person (in front of the chair) stops suddenly, we’re not stuck with a problem. The chair will stop” (61 years old, male, primary diagnosis of spinal cord injury, 39 years as a PWU). The path following function of the IPW was described as providing additional autonomy to the PWU when going to a new place or travelling long distances. 

**Table 2:** Difficulties reported with PWC and IPW features that could address these.

Challenging Situations When Using a PWC	Examples of Incidents Occurring with PWC	IPW Intelligent Function that Could Address Difficulties
*Negotiating small spaces* Negotiating narrow doorways	Scraping knuckles going through doorways Running into door frames and damaging wall or frame Scraping inside of transit vehicles Catching leg on a nail going around a corner	Obstacle avoidance/path following
*Negotiating elevator* Manoeuvring in the elevator to position PWC Backing up out of the elevator	Getting foot stuck in elevator door	Obstacle avoidance/path following
*Navigating in narrow aisles*	Breaking a store window Unintentional shop lifting	Obstacle avoidance/target following
*Driving over long distances*	Poor control of the PWC with fatigue	Path following and target following
*Navigating crowded places* (including not being visible to others)	Getting hit by a bus or car while in bike lane (using bike lane to avoid crowded sidewalks) Getting hit by a car at a crosswalk Hitting people People running into/falling on PWC/PWU	Obstacle avoidance
*Uneven/changing driving surfaces*	Falling off a curb Falling in a ditch Falling off slope in a theatre	Obstacle avoidance

Lastly, target following was reported as being useful when power mobility navigation is limited by fatigue or visual impairments. For example, one caregiver described that he takes over driving the PWC when the PWU’s ability to drive using the joystick is hindered by fatigue.

#### 3.2.2. Cautious Optimism Concerning the IPW

While participants were very enthusiastic when they saw what an IPW could do, as evidenced by their verbal expressions such as “Amazing!”, “Genius!”, and “Wow!”, they nevertheless expressed concerns on both a personal level, regarding their abilities and ways in which they would experience the IPW, as well as on a technological level. 

PWUs commented that they wanted to continue to do the tasks that they perceived themselves as currently physically able to carry out, and caregivers corroborated this finding. Participants did not want the IPW to replace the abilities of the PWU. For example, one PWU stated: “So if I have this feature, of it driving itself, I’d become, hum, really, hum I’d rely on it too much more than I need to…So it could make me lazy and not enhance my mobility” (22 years old, male, primary diagnosis of muscular dystrophy, 10 years as a PWU).

In most cases, participants perceived only some of the IPW features as relevant to them. For example, several participants were skeptical about trusting the IPW more than their instinct or their abilities, or felt they needed to be convinced about the IPW’s reliability with respect to its ability to avoid moving obstacles. For instance, one participant said, “Well for me, I…I would have difficulty trusting more a machine’s reflexes than my own abilities to control the chair. Because I have very good control of the chair. And, I think, pressing on a touch screen, waiting for it to react, that it sends a command, for me, in my head, it is faster to take my control and do what I need to do now…” (25 years old, female, rheumatoid arthritis, 10 years as a PWU). However, other participants had the opposite perspective, as described by this participant, “I wouldn’t be as quick to respond to emergency…obstacles if there’s, say, someone appears at the corner and I’m about to turn right. It could happen where I’m not quick enough to press the emergency button or to let go of the joystick, so I think it’s a plus, because this would respond I think faster than…if you’re driving by yourself” (22 years old, male, primary diagnosis of muscular dystrophy, 10 years as a PWU).

“Following a planned path” was viewed by half the participants as being useful. Those that did not find it useful expressed that this was a task that everyone has to do, for example in a shopping mall, and that this was a task they could and wanted to continue to do by themselves. 

In addition to perceived benefits of the IPW, some participants expressed concerns with some of the technical characteristics of the chair. Participants were concerned that sensors, which are used for navigation and obstacle avoidance, would increase the overall width of the chair making it more difficult or impossible to navigate in narrow spaces or go through narrow doorways, as expressed by a PWU when talking about where the sensors on the IPW, “Exactly. If we, we increase the width of the wheelchair, we just ruined many situations” (61 years old, male, primary diagnosis of spinal cord injury, 39 years PWU experience). Participants also had concerns about using the IPW for outdoor activities. They were concerned with the speed of the chair, either that it was too slow to allow them to participate in the activities in which they would like to participate, or that it may not slow down enough in the event of going over a pothole for example. They wondered whether it would be able to detect holes, edges of sidewalks, cracks in sidewalks, or other low obstacles such as cans or glass on the floor. One participant expressed the following hesitations: “Hum… that, I don’t know if I’m fully comfortable with it, (…) because sometimes like it could be something on the floor that I think it may not detect, so… let’s say, you are going towards a wall, there’s like a can on the floor.” (22 years old, male, primary diagnosis: muscular dystrophy, 10 years as a PWU). Participants also questioned how the chair would identify certain features of outdoor environments, such as red traffic lights while in intelligent mode, expressing that this would be a significant safety concern. Participants also voiced reservations with the target following feature when the target was a group. Specifically, participants wondered what happens if the group disperses, “And at some point, you don’t know anymore who you are with”. Finally, several participants questioned whether the path following ability of the IPW would work, for example, if a store would move locations, or if entering a new place where the map has not been previously loaded. 

Knowing that the IPW was still being refined, participants provided technical suggestions during the interviews. Examples of such suggestions include having an auditory signal when approaching an obstacle as a warning signal and being able to detect problems with the IPW, “… failsafe mechanism could’ve built into… hum, to warn you if hum… there is any… technical problem with it.” (65 years old, male, primary diagnosis of multiple sclerosis, 6 years as a PWU). One participant questioned how the chair would know which feature to use. He suggested that for example if the IPW is set to follow a person, it should also be able to also avoid a moving obstacle. Finally, some participants suggested that they would like to be able to choose the features they would want on their IPW if they had concerns with some features or did not consider them to be useful to them. 

#### 3.2.3. Defining the IPW User

When discussing the relevance of the different features of the IPW, all of the participants found that at least two of the features would be helpful in their activities. More specifically, most participants described situations where obstacle avoidance and path following would be useful. With the presence of fatigue, path following and target following were seen as important. Use of these features would reduce the physical and cognitive demand involved in navigating the PWC, thereby increasing independence for the PWUs and decreasing caregiver burden for those caregivers who often take over driving the PWC in these situations. The husband of a PWU participant with multiple sclerosis described several situations in which he currently helps his wife and felt confident he would not have to do so with an IPW, “For example, I, I imagine that I would not have to sometimes move the WC to make more room, she sometimes parks it at a certain distance…” and “… I would not always have to accompany her, hum…., when she goes out if she wants to go out…to a shopping mall.” (Caregiver, 57 years old). 

PWU and caregivers identified characteristics of people who would likely benefit from the IPW, including poor upper extremity motor control, decreased orientation, poor reaction times, decreased vision, and fatigue. They also suggested that the IPW becomes more relevant as people age, increased cognitive and visual impairments. For example, one PWU stated “…for orientation, that is, that is interesting, when I was talking about cognitive difficulties… and that you want to go out alone…because often when you have cognitive difficulties you will be accompanied by someone to go and do things. But here, (the person) could be alone. And for elderly people, exactly, the fact that they can follow a person or hum….it avoids maybe making a wrong move, because when you are with someone, you tend to be close to them….and you make a wrong move the other person trips on your WC….so it would be more reassuring pour elderly people or also for people who have problems with motor abilities” (45 years old, female, primary diagnosis of multiple sclerosis, 3 years as a PWU).

Overall, the IPW would be advantageous for those individuals with decreased autonomy related to their power wheelchair use.

Some participants emphasized how the IPW features would become more useful as their condition progressed. For example, two participants with multiple sclerosis, could see the benefit of the IPW should their condition deteriorate, in particular with respect to loosing upper extremity control or having increased fatigue, as indicated by a PWU talking about using the IPW to avoid obstacles instead of manually controlling his current PWC, “A priori, it would not be now, in the sense that now I still have good use…but absolutely, it would be something that would need to be considered yes yes, absolutely.” (44 years old, male, primary diagnosis of multiple sclerosis, 7 years as a PWU). Conversely, another participant, with a very slow progressive spinal muscular atrophy, did not feel that his condition was likely to deteriorate to the point of losing motor control in the hands and hence did not feel he would need to use the IPW. 

When questioned about their intent to use the IPW if it were available today, half of the PWUs expressed that they would use it, and three of the four caregivers expressed that the PWU for which they provide care would likely use it. Some even expressed that they would feel safer (or in some cases people around them would be safer) if they were driving an IPW rather than their current PWC. Those that did not feel that they would use it today explained that they found it less relevant for them because of their current capabilities, although they could identify other PWUs who would benefit from it.

## 4. Discussion

In this article, we have described the perceptions of PWUs and caregivers regarding IPW use. The results of this study indicate that PWUs and their caregivers encounter challenging situations with their current PWC that may be overcome by the IPW. In fact, the IPW may not only eliminate the need for certain compensatory strategies (e.g., avoiding crowded venues), it may also reduce safety hazards associated with other strategies (e.g., driving the power wheelchair in bike and bus lanes). However, while there are clear benefits to the use of the IPW, participants also raised important questions and concerns from both a personal and technological perspective. Through the user-centered approach implemented in this study, the results provide important information for the further development of IPWs. Impact that IPWs may have on difficulties encountered by PWUs and recommendations for future design are discussed in the following section.

### 4.1. Impact of IPWs on Current Challenges Faced by PWUs

To date, most studies in the field of IPWs have focused on developing and testing the intelligent features of the IPWs. For example, Nguyen *et al.* [23] reported on the use of an IPW with a brain-computer interface or a head movement controller with eight able-bodied individuals and two people with tetraplegia. They analyzed path navigation and reported decreased time to complete an obstacle course with the brain-computer interface coupled with the intelligent PWC. How *et al.* [24] assessed the usability, efficacy and safety of an add-on for a PWC to assist with navigation and obstacle avoidance in two cognitively impaired individuals. They reported that the WC avoided collisions and was able to navigate as intended, while decreasing the perceived demands of the tasks by the users. They also reported user satisfaction, which tended to be greater with the add-on than without. Montesano *et al.* [25] evaluated the use of an IPW designed for children with cognitive and motor impairments. They tested driving performance of the IPW with four children with cerebral palsy and observed user interest and enthusiasm during the tasks. There is no doubt that studies examining IPW capabilities are essential. However, along side these studies, there is a paucity of studies reporting user perceptions regarding IPWs.

In our study, we interviewed PWUs’ and caregivers’ regarding IPW use. First we obtained a portrait of the challenges participants face with their current PWC, including accidents or near-misses that occurred indoors and outdoors, such as when backing up, navigating in crowded places, and avoiding low lying obstacles and holes. Similar challenging situations were reported by Wang et al [26], who interviewed mobility device users over the age of 65, caregivers and therapists about a collision avoidance technology for a PWC. In their study, the authors exclusively addressed the obstacle avoidance function as this was the main intelligent of the IPW they had developed, and it was found to be perceived as less useful for avoiding dynamic obstacles. Participants in our study expressed that the all the IPW features, including obstacle avoidance, path following and target following could help overcome some of the challenges reported. The differences with regards to obstacle avoidance may in part be due to the methods used for soliciting feedback. In our study we used a 4-min video illustrating each of the intelligent features in a mall setting, including avoiding dynamic obstacles such as people, where as Wang *et al.* [26] provided a verbal description of the collision avoidance technology. Hence, the participants’ perception may in part be influenced by their understanding of the IPWs abilities and limitations. 

Some participants acknowledged that if the PW were available now they would not choose to use the IPW. Participants reported similar reasons to those reported by Wang *et al.* [26], namely feeling that they may have better driving abilities than an automated wheelchair, not wanting to rely on the IPW to do what they feel they can do themselves, or lacking confidence in the IPWs abilities. In addition, some participants in our study were concerned that the speed of the IPW may be too slow for their activities. This was also reported in the two small studies which documented IPW perceptions of elderly residents of long term care facilities [27,28]. Taken together, these findings underscore the importance of understanding and taking into account the needs of eventual IPW drivers. 

It should be noted that these types of studies provide a snapshot at one point in time, of an innovative technology that is not yet commercially available. Hence, over time, users’ perspectives may evolve as newer technologies, such as technologies using GPS capabilities and collision avoidance, become more routinely used in society in general, which may in turn change how these technologies are perceived by potential IPW users. 

### 4.2. Design Recommendations for the IPW Using a User-centered Approach

As suggested by user-centered approaches for design, our research team has continued to integrate the PWU’s perspective at pivotal points in the development of the IPW. For example, at a more preliminary stage of development, user input led to the inclusion of a tactile control panel to reduce difficulties encountered by the PWU with a vocal interface [29], and IPW use was assessed using an earlier prototype in a controlled setting [22]. The current study provides insight, from the PWU and caregiver’s perspective, which can then be used to further inform development of the current and future IPWs. Participants provided information about challenging situations encountered with their current PWC, both indoors and outdoors, some of which could potentially be overcome with an IPW (Table 2). Additional described challenges, as well as voiced questions, concerns, and feedback have provided our research team with design ideas for the continued development of the IPW. Future refinements of IPW prototypes should take participants’ concerns and feedback for improvement, such as the one listed in Table 3, into account. 

**Table 3:** Design recommendation examples for IPWs based on participants’ feedback.

Participants’ Feedback	Design Recommendations for the IPW
The IPW should be able to detect holes, edges of sidewalks, cracks in sidewalks, or other low obstacles such as cans or glass on the floor.	Detect “negative” objects, such as edges of curbs and ramps, docks, potholes, stairs Intelligent speed adjustment to account for changes in driving surface, such as cracks and potholes in sidewalks Detection of low lying obstacles, such as glass
The IPW should be able to go at a speed needed to participate in the desired activities	Speed control options in intelligent mode
How will the IPW identify certain features of outdoor environments, such as red traffic lights while in intelligent mode?	Detection of traffic signals in intelligent mode, such as red lights and walk/don’t walk signals
Not all the features are relevant to everyone	Provide PWU with the ability to opt in or out of particular IPW features
The sensors on IPW may increase the overall width of the chair making it more difficult or impossible to navigate in narrow spaces	Minimize increases in PWC width and length secondary to sensors
There should be a mechanism to indicate if the IPW is not functioning correctly	Provide indicator signal for system failures, such as computer, motor or sensor malfunction
There should be a signal warning when approaching an obstacle	Include optional audio alert for obstacle avoidance intelligent feature option

When designing assistive technologies for people with disability, a significant challenge is designing a technology in a group that presents a range of abilities [20]. Indeed, in this study, while participants found at least two of the functions of the IPW relevant for their use, several participants considered that at least one of the functions would not be of use to them. Knowing that some but not all the IPW features are appropriate for everyone, refinement of the prototype could therefore allow users to select the options appropriate for them, as suggested by some participants. 

Participants in this study had a number of questions about the way in which the IPW would function, in particular with respect to the path-finding and following a group functions. This emphasizes the need for adequate training and education of future IPW users when the IPW is ready to be used by PWUs in a real setting, in order to address and alleviate concerns they may have which may limit their use of the IPW. 

### 4.3. Study Limitations and Future Directions

This study has several limitations. First, the participants provided feedback on the IPW based on a video rather than personal experience in a real-life context. Hence, we do not know if and how their perspectives regarding the IPW would have been different had they actually experienced the IPW. Nevertheless, our results suggest that the video was effective in facilitating participants’ recall of personal situations similar to those depicted in the video. Further, the video also allowed participants to recall problematic situations they had encountered which had not been raised prior to viewing the video as well as situations not depicted in the video, such as outdoor and other community-based activities. Hence, the use of the video did not limit the scope of activities recalled by our participants. Therefore, in our opinion, the video is a useful tool to document perceptions regarding IPWs at this point in time, when actual independent use in real environments is not yet possible. 

Another limitation of this study is that only the perspectives of current PWUs were elicited. Future studies could include individuals who are not currently using a PWC, both those who have been denied use (e.g., secondary to safety concerns), as well as those who are planning to obtain a PWC. Perspectives of clinicians will also be important to explore in terms of understanding for whom an IPW would or would not be prescribed. In addition, only four caregivers were recruited in this study. For this study, we were looking to include caregivers who assist their close ones for mobility tasks, in order to document their perspectives on the use of an IPW and the impact it might have on the PWU or themselves. However, despite recruiting efforts in two large wheelchair and seating departments as well as through the PWUs, few people met this criteria. Future studies could include the perspective of more formal caregivers who may be more involved in assisting PWUs in mobility tasks. For this study, we did not conduct member checking with participants after the interviews. 

A convenience sample of PWU and caregivers was used for this study, which may limit generalizability of the results to the larger PWU population. Nevertheless, the participants in our study do reflect the age range and diversity of health conditions of non-institutionalized individuals who use power mobility devices, as reported by the National Institute on Disability and Rehabilitation Research reporting on United States census data from 1994 [30]. In addition, our sample did not include participants with cognitive deficits. Although some of the current IPWs being developed are aimed at this patient population, the IPW developed by our team is in fact not designed for PWU with cognitive impairments.

In qualitative research, one aspect of internal validity is the credibility of the data collected [31,32]. It is important to consider the interviewer’s perspective and its possible impact on the data collected. In this study, the interviewer's extensive knowledge of and experience with power wheelchairs and the wheelchair community may be seen as a limitation in that the participants may have responded to the interview questions positively in order to please this occupational therapist who has contributed to their community in many ways. However, it was also this knowledge and experience that enabled the study interviewer to quickly and easily develop rapport with the participants, understand their described experiences and situations, and effectively probe in order to acquire our rich data. 

Findings from this study cannot be generalized to other settings which may have different geographical and contextual realities. Participants in this study lived in Montreal, Quebec, Canada, a city with a northern climate with harsh winters often leading to poor road conditions, a factor which was raised numerous times during the interviews. In addition, wheelchairs are prescribed and reimbursed within a public health care system. Participants in this study did not address the issue of cost as a barrier to using the IPW, and this may in part be due to the fact that once a wheelchair is prescribed and approved its cost is covered by a provincial Medicare program. This type of prescribing and funding system may not be the case in other geographical locations 

Interestingly, our results suggest that the IPW, when used in a social context, could have potential for change in social participation. For example, participants described how driving in an IPW could change the way in which they experience activities such as going to the mall with their spouse. Further analyses are currently underway to better understand how IPWs could impact social participation. In addition, future studies should include the development of appropriate measurement tools and methodologies that will be used to assess actual IPW use in a variety of natural settings. For example, our team is currently validating an existing measure of wheelchair navigation, the WheelChair Skills Test 4.1 for use with an IPW in a shopping center environment. 

## 5. Conclusions

Including the key stakeholders’ perspective in the design and development process of the IPW is essential. It allows early detection of potential challenges and obstacles. Using an iterative process as proposed here, the stakeholders’ input can be integrated into the IPW during the development phase. Future studies exploring the prescribers’ perspective, as well as evaluating actual use with PWUs and other potential IPW users will be essential to continue to develop an IPW that best meets the users’ needs, and thus increasing community participation.
